# Salinity-driven nonlinear responses of microbial functional genes in sediment biogeochemical cycling across a salt lake gradient

**DOI:** 10.1128/aem.00721-26

**Published:** 2026-06-04

**Authors:** Mingxian Han, Jianrong Huang, Jian Yang, Qing Liu, Chuanxu Wang, Xin Li, Hongchen Jiang

**Affiliations:** 1 School of Life Sciences, Henan University 12411, Kaifeng, China; 2 College of Life Sciences, Yuncheng University 92265, Yuncheng, China; 3 Shanxi Key Laboratory of Yuncheng Salt Lake Ecological Protection and Resource Utilization, Yuncheng University 92265, Yuncheng, China; 4 Department of Biology, Xinzhou Normal University 66353 , Xinzhou, China; Colorado School of Mines, Golden, Colorado, USA

**Keywords:** salinity gradient, microbial functional genes, biogeochemical cycling, salt lake sediments, microbial community assembly

## Abstract

Salinity is a major environmental driver in saline lake ecosystems, yet the functional genes responses underlying sediment biogeochemical cycling remain poorly resolved. Here, we investigated microbial community composition and functional gene dynamics along a pronounced salinity gradient in Yuncheng Salt Lake. Salinity strongly restructured microbial assemblages, producing distinct taxonomic groups dominated by *Actinobacteriota*, *Gammaproteobacteria,* and *Halobacteria* across low-, moderate -, and high-salinity sediments. Stochastic processes governed community assembly at low and high salinity, whereas deterministic selection increased at moderate salinity. Functional genes associated with carbon, nitrogen, phosphorus, and sulfur cycling exhibited nonlinear, pathway-specific shifts in diversity and abundance, reflecting differential sensitivities to salinity. Differentially abundant genes and the distribution of shared vs unique functions further highlighted strong salinity-driven restructuring of microbial functional potential. Microbial community dissimilarity was tightly coupled to functional gene divergence, and environmental analyses identified salinity as a dominant abiotic regulator, supplemented by influences of pH, TOC, TN, and DOC. Structural equation modeling revealed that salinity regulates functional gene abundance both directly and indirectly via strong effects on microbial diversity. Overall, these findings demonstrate that salinity shapes sediment biogeochemical potential through coordinated impacts on microbial community structure and functional gene organization.

**IMPORTANCE:**

Salinization is increasing globally in inland waters and can substantially alter microbial processes that regulate sediment biogeochemistry. However, how microbial functional genes governing carbon, nitrogen, phosphorus, and sulfur cycling respond to salinity gradients remains poorly resolved. By examining microbial communities and functional genes across a natural salinity gradient in salt lake sediments, this study demonstrates that salinity not only restructures microbial communities but also drives nonlinear changes in functional gene assemblages. The strong association between taxonomic turnover and functional gene divergence indicates that shifts in microbial community composition are closely linked to changes in functional potential. Identifying salinity as the dominant driver of functional gene organization provides new insights into how salinization may influence microbially mediated biogeochemical processes in saline environments. Together, these findings enhance our understanding of microbial functional dynamics in salt lake sediments and offer a framework for predicting ecosystem responses to ongoing environmental salinization.

## INTRODUCTION

Understanding how microbial communities respond to environmental change is a central challenge in microbial ecology, with important implications for ecosystem functioning and biogeochemical processes (1–3). Microorganisms play key roles in regulating the transformation and cycling of carbon (C), nitrogen (N), phosphorus (P), and sulfur (S) (4), and the distribution of microbial functional genes provides an important link between community composition and ecosystem processes (5–7). In natural habitats, microbial functional potential is shaped by the interaction between environmental filtering and biotic interactions, which together determine community structure and metabolic capability (8–10). Among environmental factors, salinity represents one of the most influential selective forces affecting microbial life (11). Elevated salinity imposes strong osmotic stress, modifies nutrient availability, and can profoundly restructure microbial communities (12–15). While numerous studies have documented taxonomic shifts in microbial communities along salinity gradients (16–19), the responses of functional genes associated with key biogeochemical processes remain underexplored, particularly across broad gradients spanning brackish, saline, and hypersaline conditions.

Microbial functional stability is often attributed to functional redundancy, whereby phylogenetically distinct taxa perform similar ecological roles, thereby buffering ecosystem processes against environmental perturbations (20–22). However, the strength of this buffering capacity under strong environmental stress remains uncertain. Salinity simultaneously affects microbial physiology, resource availability, and community composition, potentially reshaping the distribution and abundance of functional genes associated with elemental cycling (23, 24). Moreover, functional genes involved in different biogeochemical pathways may respond differently to salinity stress due to variation in metabolic requirements and ecological niches (25, 26). As a result, shifts in microbial functional potential may not necessarily follow simple linear trends along environmental gradients.

Despite increasing attention has been given to microbial responses to salinity, most studies have focused primarily on taxonomic diversity (27, 28), with comparatively less attention given to how functional genes associated with C, N, P, and S cycling respond across broad salinity gradients. In particular, pathway-specific responses of these functional genes remain poorly characterized in saline lake sediments, especially across transitions from brackish to hypersaline conditions (29). Furthermore, microbial functional gene dynamics may exhibit nonlinear responses to environmental gradients, reflecting differences in ecological strategies, physiological tolerances, and resource availability among microbial taxa occupying distinct metabolic niches (30, 31). Elucidating how microbial functional genes respond to salinity gradients is, therefore, essential for understanding microbial contributions to biogeochemical cycling in changing environments.

Another key challenge is disentangling the relative roles of abiotic and biotic factors in shaping microbial functional potential (32). Environmental variables such as salinity, nutrient availability, and pH can directly influence microbial metabolism and gene expression (33–35), while microbial diversity and community composition may indirectly regulate functional potential through the distribution of metabolic capabilities within microbial assemblages (36, 37). Understanding the relative contributions of these abiotic and biotic drivers is critical for explaining how microbial functional gene assemblages are structured in saline ecosystems.

In order to fill the above knowledge gaps, we hypothesized that (i) salinity acts as a dominant environmental filter structuring microbial community composition and functional gene assemblages and (ii) microbial functional genes involved in C, N, P, and S cycling exhibit nonlinear and pathway-specific responses to salinity due to combined environmental filtering and community turnover. The specific objectives of this study were to (i) quantify the relative roles of deterministic vs stochastic processes in assembling microbial communities under varying salinity regimes; (ii) characterize the linear and nonlinear responses in the abundance and diversity of key C, N, P, and S cycling genes; and (iii) disentangle the direct effects of salinity on functional potential from its indirect effects mediated through changes in microbial diversity and community composition. By integrating taxonomic profiling and high-throughput functional gene quantification, we explored salinity patterns of the six subgroups of functional genes and investigated the relative importance of abiotic and biotic factors in explaining their diversity and composition across a pronounced salinity gradient in Yuncheng Salt Lake, Shanxi, China.

## MATERIALS AND METHODS

### Site description and sediment sampling

Sampling and on-site measurements were conducted at Yuncheng Salt Lake in early September 2024. Sediments were collected from eight sites distributed across a broad geographical and physicochemical gradient within this closed inland saline system, which has experienced long-term evaporation and salt extraction that resulted in multiple hydrologically isolated ponds exhibiting distinct water colors and salinity regimes (38). The lake covers approximately 120 km^2^ and is characterized by an annual mean temperature of 13–14°C, mean precipitation of about 550 mm, and evaporation exceeding 2,000 mm (39, 40). Eight representative ponds labeled Sed_A to Sed_G were selected along a natural and anthropogenic salinity gradient ranging from 20.08 to 259.60 g L^−1^ as shown in Fig. S1. GPS coordinates were recorded using a portable GPS unit (eTrex H, Garmin, USA). At each site, surface sediments (1–5 cm depth) were collected in triplicate with sterile spatulas that had been cleaned and rinsed with alcohol and ultrapure water. Samples intended for microbial DNA extraction, high-throughput quantitative PCR and physicochemical analyses were sealed in sterile bags, stored in refrigerated containers, and transported on ice to the laboratory for processing within 24 h.

### Microbial DNA extraction and amplicon sequencing workflow

Total genomic DNA was extracted from approximately 0.5 g of sediment using the Fast DNA SPIN Kit (MP Biomedicals, Santa Ana, CA, USA) following established procedures (11, 41). The hypervariable V4 region of the microbial 16S rRNA gene was amplified using primers 515F (5′-GTGYCAGCMGCCGCGGTAA-3′) and 806R (5′-GGACTACNVGGGTWTCTAAT-3′) (42), with each primer set incorporating a unique barcode for multiplex sequencing. PCR amplification was performed in triplicate in 50 μL reactions containing 100 ng of purified DNA under the following conditions: initial denaturation at 94°C for 3 min, 35 cycles of denaturation at 94°C for 45 s, annealing at 50°C for 60 s and extension at 72°C for 90 s, and a final extension at 72°C for 10 min. Amplicons of approximately 400 bp were pooled, gel-purified using a DNA Gel Extraction Kit (Axygen, USA), and sequenced using a 2 × 250 bp paired-end strategy on an Illumina HiSeq 2500 platform according to the manufacturer’s instructions.

Raw sequences were processed using cutadapt v3.5 to trim primers and remove reads shorter than 200 bp. Subsequent bioinformatic analysis was performed in DADA2 package v1.18 (43). This included quality filtering (removing reads with ambiguous bases, expected errors > 2, or quality scores < 10), model-based error correction, and dereplication. Dereplicated paired-end reads were merged with zero mismatches and a minimum overlap of 50 bases, and chimeras were removed using the consensus method. Taxonomic assignment of microbial amplicon sequence variants (ASVs) was conducted with the naïve Bayesian classifier against the SILVA 138.1 database (44). Sequences assigned to chloroplasts, mitochondria, or unclassified at the domain level were discarded. To ensure comparability, samples were rarefied to the minimum sequencing depth (66,224 reads per sample) using the *rrarefy* function in the R package “vegan” (45) to ensure comparability across samples.

### High-throughput quantification of microbial functional genes

Microbial functional traits were characterized using the Quantitative Microbial Element Cycling (QMEC) functional gene array based on high-throughput quantitative PCR (qPCR) (46), utilizing the 16S rRNA gene as an internal reference. A total of 71 functional genes involved in carbon, nitrogen, sulfur, and phosphorus cycling were quantified using the SmartChip Real-Time PCR system. Each sample was analyzed with three technical replicates and included negative controls. Genes were considered positively detected if the negative control showed no amplification, amplification efficiency ranged from 1.8 to 2.2, and the Ct value was <31. The copy number of each target gene per gram of sediment was subsequently calculated based on the Ct values and corresponding 16S rRNA gene copy numbers, following established methods (32, 47). Notably, extreme salinity in certain samples inhibited extraction and amplification efficiencies, falling below the reliable detection limit of the high-throughput qPCR assay. Consequently, these hypersaline samples (Sed_A, Sed_B, Sed_C_Yellow, Sed_C_Pink, Sed_D; salinity range 129–260 g/L) were excluded from downstream analyses; functional gene profiles are reported only for the brackish (Sed_G, salinity 21 g/L), saline (Sed_F, salinity 75 g/L), and hypersaline (Sed_E, salinity 158 g/L) groups that remained within the quantifiable range.

### Environmental and microbial variables

In the laboratory, sediment porewater was extracted by centrifugation at 12,000 × *g* for 10 min. Porewater pH was measured with a portable multiparameter instrument (SANXIN, Shenyang, China), and major cations and anions including Li^+^, Na^+^, NH_4_^+^, K^+^, Mg^2+^, Ca^2+^, F^−^, Cl^−^, NO_3_^−^, NO_2_^−^, SO_4_^2−^, Br^−^, and PO_4_^2−^ were quantified in sediments porewater using ion chromatography (Dionex DX-600, Sunnyvale, CA, USA). Porewater salinity was calculated as the sum of measured major ions in grams per liter. Dissolved organic carbon (DOC) and total organic carbon (TOC) were analyzed by high-temperature catalytic oxidation coupled with nondispersive infrared detection using a multi N/C 2100S analyzer (Analytik Jena, Germany). Prior to dissolved organic carbon analysis, samples were filtered through 0.45 μm cellulose acetate membranes and acidified with concentrated phosphoric acid, whereas unfiltered aliquots were used for total organic carbon determination. Dissolved inorganic carbon (DIC) was measured by potentiometric acid titration, and saturated mercury chloride was added to each vial before sealing (48). Total phosphorus (TP) and total nitrogen (TN) were quantified colorimetrically following established protocols (49, 50). The detailed physicochemical profiles of sediment samples across all sites were presented in Table S1. Biotic attributes of microbial communities were represented by Shannon diversity, which quantified α diversity, and by the first two principal coordinates derived from principal coordinates analysis (PCoA), which summarized variation in community composition among samples.

### Statistical analyses

Statistical analyses were conducted in R version 4.5.1 using a suite of specialized packages. Alpha diversity (species richness and Shannon index) was calculated using the “vegan” package (v2.6-4). Treemaps were constructed using the “voronoiTreemap” package (v0.2.1) to visualize the relative abundance of major microbial phyla across salinity ranges, while differentially abundant ASVs among salinity groups were identified through volcano plots based on DESeq2 (v1.42.0) analysis. Variation in microbial community structure was visualized using PCoA (based on Bray-Curtis distances) and tested for significance using PERMANOVA (Adonis2 test, 999 permutations). Community assembly processes were quantified using null model analyses, where the relative contributions of deterministic and stochastic processes were inferred from the beta nearest taxon index (βNTI) and Raup-Crick metric (RCbray) computed with the “microeco” package (v1.2.0) (51).

For functional gene analysis, Sankey diagrams generated with the "networkD3" package (v0.4) visualized compositional shifts of key functional genes across salinity gradients, while heatmaps created via the "pheatmap" package (v1.0.12) displayed the relative abundance of functional gene categories across samples. Venn diagrams (52) illustrated the shared and unique genes among different salinity groups, and shared genes networks were constructed using the “ggraph”" package (v2.1.0). Relationships between absolute functional gene abundance and environmental variables were examined using locally estimated scatterplot smoothing (Loess) smoothing for trend fitting (53). To assess whether nonlinear models better captured salinity-gene abundance relationships than linear models, we compared Akaike Information Criterion (AIC) values between loess and linear regression fits for each biogeochemical pathway. Lower AIC values indicate superior model fit, with a difference (ΔAIC) >2 considered meaningful support for the nonlinear model. To link community composition with functional structure, Bray-Curtis community distances were regressed against functional gene distances using the “ggplot2” (v3.5.1) and “ggpmisc” packages (v0.5.6). Pairwise associations between functional genes, environmental variables, and microbial metrics were assessed using correlation heatmaps implemented with the “corrplot” package (v0.92). Environmental drivers were further quantified using linear regression between functional gene distance and physicochemical variables, and distance-based redundancy analysis (dbRDA) with variance partitioning quantified the explanatory contributions of environmental factors (54). Finally, structural equation modeling (SEM) implemented in the “piecewiseSEM” package (v2.3.1) (55) tested hypothesized causal pathways among salinity, microbial diversity, functional diversity, and functional gene abundance, with standardized effect sizes calculated for significant paths.

## RESULTS

### Salinity restructures microbial community composition and assembly processes

**Fig 1:**
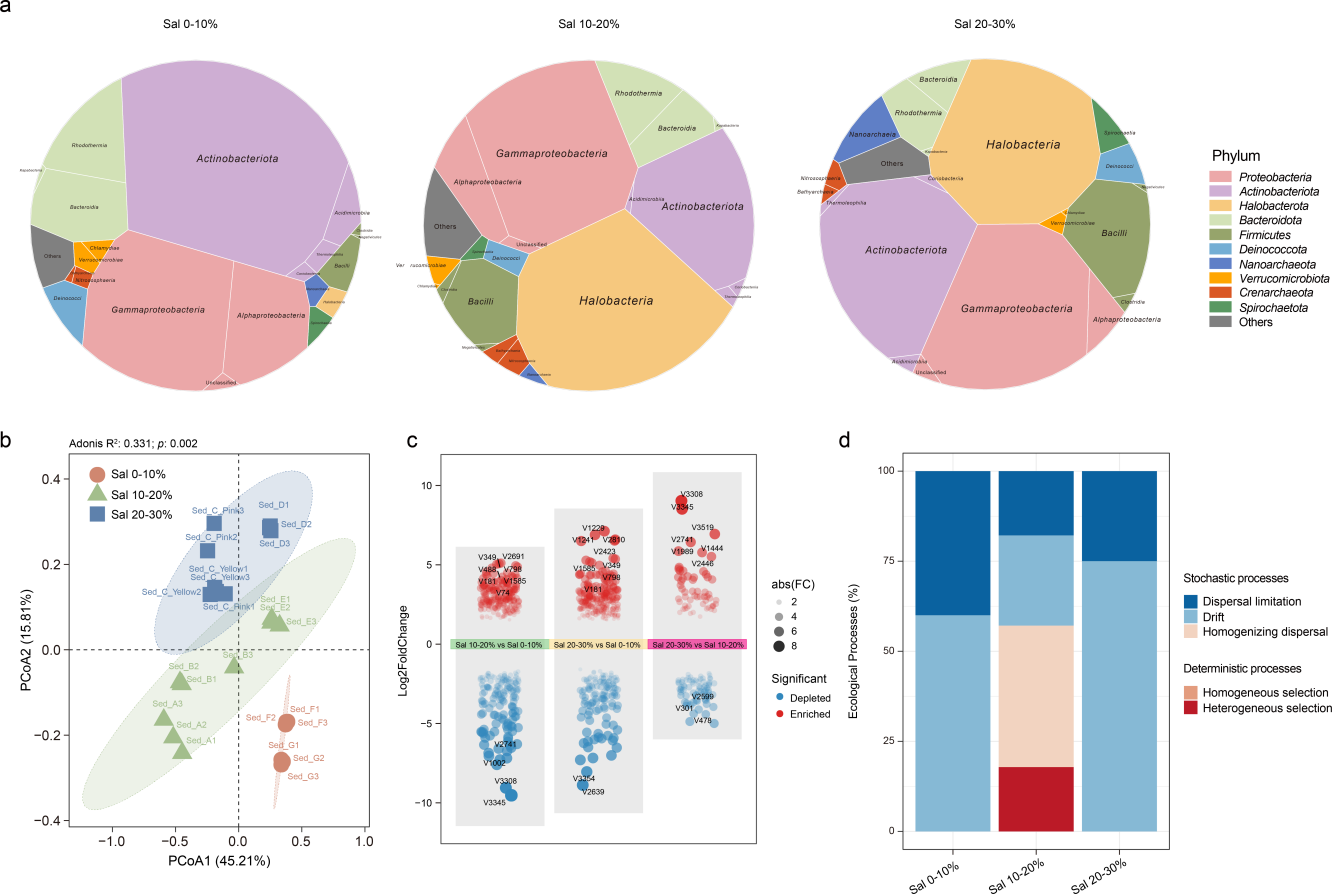
Microbial community composition and assembly mechanisms across a salinity gradient in lake sediments. (**a**) Relative abundance of major microbial phyla and classes at three salinity ranges. (**b**) Principal coordinates analysis (PCoA) based on Bray-Curtis dissimilarities, showing significant separation of communities along the salinity gradient. (**c**) Volcano plots identifying differentially abundant ASVs among salinity groups. (**d**) Relative contributions of deterministic and stochastic processes to community assembly under different salinity regimes.

Salinity markedly restructured microbial communities in the sediments, driving distinct taxonomic successions in taxonomic compositions across salinity ranges (Fig. 1a). The dominant microbial lineages shifted from a prevalence of *Actinobacteria*, *Gammaproteobacteria,* and *Alphaproteobacteria* in the Sal 0%–10% group, to a clear dominance of *Gammaproteobacteria* and *Halobacteria* in the Sal 10%–20% and Sal 20%–30% group. This compositional turnover was corroborated by principal coordinates analysis, which revealed significant separation of communities among the three salinity groups (Adonis2, *R*^2^ = 0.331, *P* = 0.002; Fig. 1b). Accordingly, volcano plots identified numerous differentially abundant ASVs between salinity groups (Fig. 1c), supporting extensive taxonomic turnover along the salinity gradient. The relative influence of ecological assembly processes also varied with salinity (Fig. 1d). Stochastic processes dominated across all salinity groups and were the sole drivers in the Sal 0%–10% and Sal 20%–30% groups, where ecological drift contributed 60% and 75%, and dispersal limitation 40% and 25%, respectively. In the Sal 10%–20% group, stochastic processes still prevailed (homogeneous dispersal 39.29%, dispersal limitation 17.86%, ecological drift 25%), accounting for 82.14% of assembly, while heterogeneous selection (17.86%) represented the only notable deterministic force. These results indicate that increasing salinity not only reorders microbial community composition but also modulates the balance between stochastic and deterministic assembly mechanisms.

### Salinity-driven nonlinear responses of microbial functional genes

**Fig 2:**
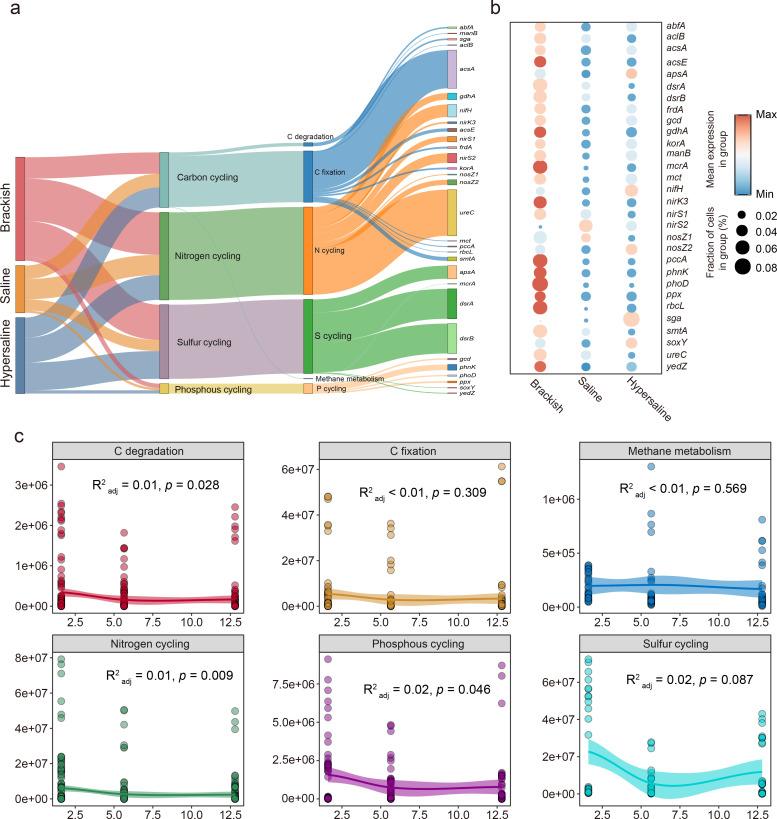
Nonlinear responses of microbial functional genes to salinity gradients. (**a**) Sankey diagram showing the distribution of the top 30 functional genes involved in carbon (C), nitrogen (N), phosphorus (P), and sulfur (S) cycling across brackish, saline, and hypersaline sediment groups. (**b**) Heatmap displaying mean normalized abundances of key functional genes, with hierarchical clustering showing salinity-dependent patterns. (**c**) Loess regression curves depicting nonlinear relationships between salinity and functional gene abundance for key biogeochemical pathways, highlighting pathway-specific responses.

Salinity gradients triggered pathway-specific variation in microbial functional potential associated with biogeochemical cycling. The Sankey diagram (Fig. 2a) illustrated the differential allocation of the top 30 functional genes across brackish, saline, and hypersaline compartments, with distinct fluxes for carbon (C), nitrogen (N), phosphorus (P), and sulfur (S) cycling processes, indicating a salinity-driven divergence in functional gene distribution. The heatmap of mean normalized gene abundances (Fig. 2b) further demonstrated clear clustering of functional gene profiles by sediment groups, supporting a salinity-dependent restructuring of microbial functional potential. Salinity also affected the α-diversity of functional genes (Fig. S2). Functional gene Shannon diversity was significantly lower in saline sediments compared with brackish and hypersaline groups, while observed richness decreased only in hypersaline sediments. Loess regression analyses, supported by model comparison using AIC (Table S2), revealed divergent response patterns of functional gene abundances to the salinity gradient (Fig. 2c). Model comparison using AIC indicated that loess models provided a better fit than linear models for all six pathways (Table S2). Significant but relatively weak trends were detected for C degradation, N cycling, and P cycling (*P* < 0.05, adjusted *R*^2^ = 0.04–0.11). In contrast, genes involved in C fixation, methane metabolism, and S cycling exhibited showed stronger nonlinear no significant relationships with salinity (*P* > 0.05). These results highlight heterogeneous nonlinear dynamics of microbial functional genes underlying key sediment biogeochemical processes.

**Fig 3:**
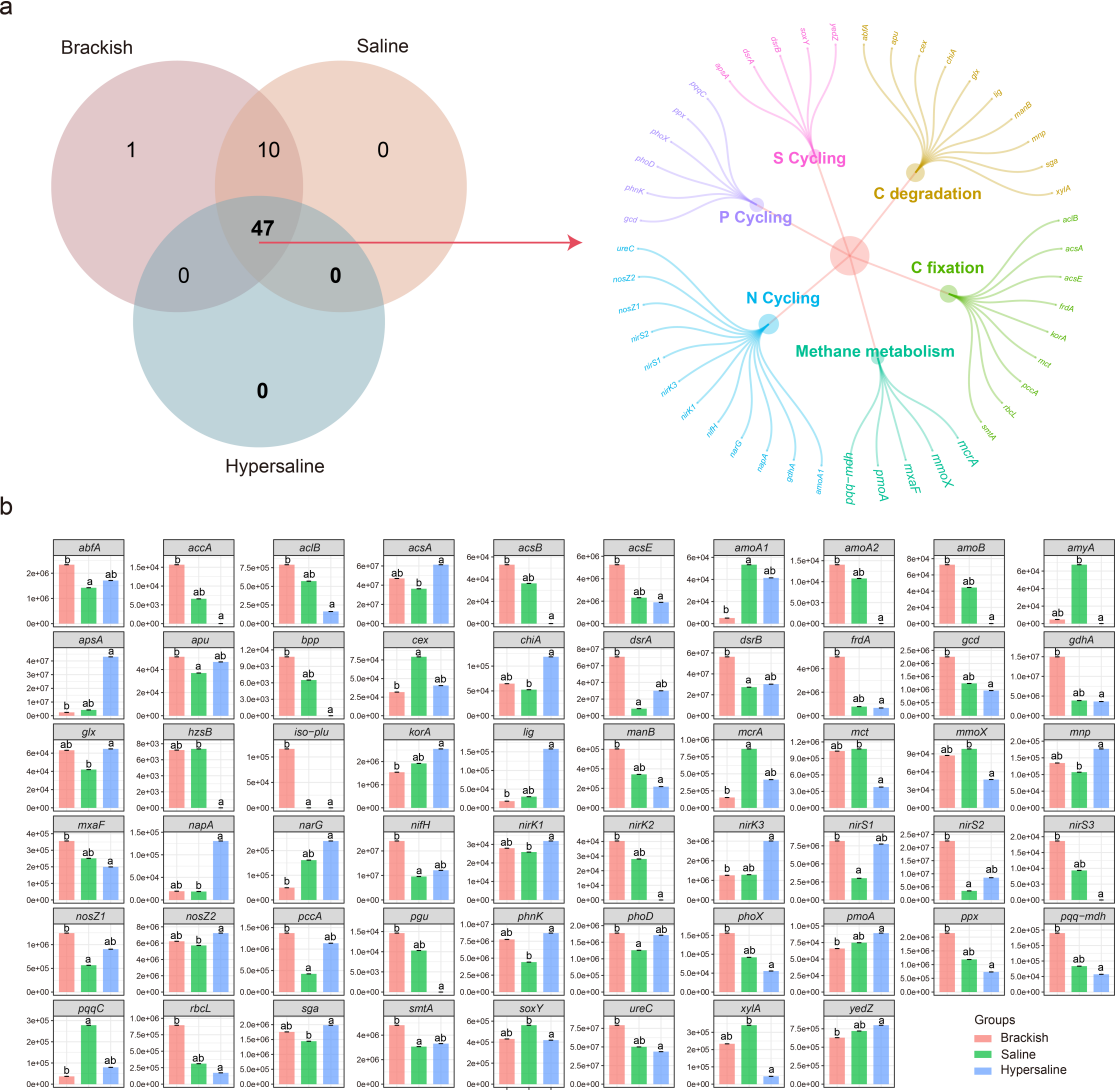
Shared and unique functional genes across the three salinity groups. (**a**) Venn diagram illustrating the number of functional genes common or unique to brackish, saline, and hypersaline sediments. (**b**) Differential abundance profiles of functional genes significantly enriched or depleted in each salinity group.

Salinity gradients also influenced the distribution of shared and unique microbial functional genes (Fig. 3a). A total of 47 functional genes were shared among brackish, saline, and hypersaline sediments. Brackish and saline sediments contained 1 and 10 unique genes, respectively, whereas no unique genes were detected in hypersaline sediments. These shared genes were associated with multiple biogeochemical pathways, including carbon degradation and fixation, methane metabolism, and N, P, and S cycling. Differential abundance analysis revealed substantial salinity-dependent variation in functional gene distributions (Fig. 3b). In carbon cycling, *accA* (involved in C fixation) was enriched in brackish sediments, whereas *pmoA* (related to Methane metabolism) showed higher abundance in hypersaline sediments. Within nitrogen cycling, *amoA*2 (encoding archaeal ammonia monooxygenase subunit A) was more abundant in brackish sediments, whereas *amoA*1 (encoding bacterial ammonia monooxygenase subunit A) was enriched in saline sediments. Genes involved in phosphorus cycling (e.g., *pqqC*) and sulfur cycling (e.g., *dsrA*) also exhibited distinct salinity-dependent abundance patterns. Collectively, these results demonstrate salinity-driven nonlinear variation in the abundance of functional genes underlying key biogeochemical processes.

### Environmental drivers of microbial functional gene divergence across salinity gradients

**Fig 4:**
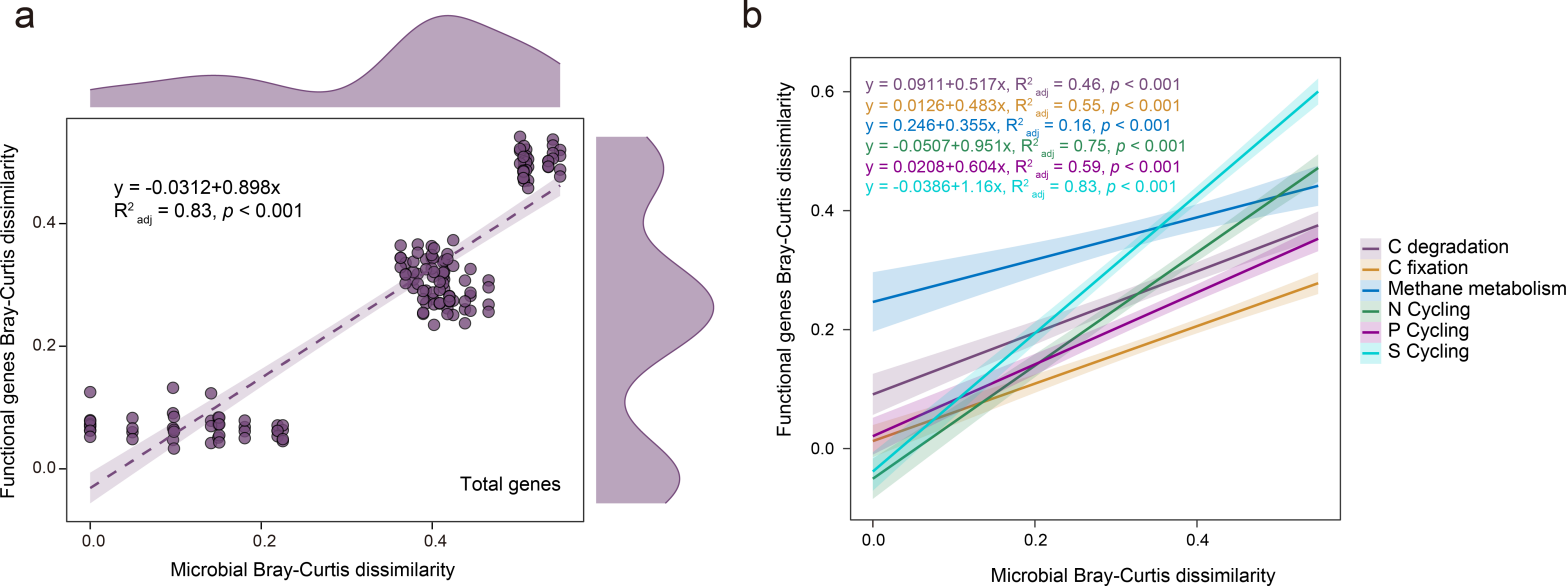
Coupling between microbial community dissimilarity and functional gene divergence across salinity gradients. (**a**) Liner analysis showing correlations between community composition distance (ASV-level Bray-Curtis) and functional gene distance. (**b**) Co-occurrence network between ASVs and functional genes demonstrating how taxonomic divergence translates into functional gene differentiation under varying salinity regimes.

Microbial community differentiation was closely associated with functional gene divergence along the salinity gradient. Linear regression analysis revealed a significant positive correlation between microbial community Bray-Curtis dissimilarity and functional gene dissimilarity (*R*^2^_adj_ = 0.83, *P* < 0.001) (Fig. 4a). This close coupling was consistent across individual biogeochemical pathways (C degradation, C fixation, methane metabolism, N cycling, P cycling, and S cycling), all of which showed were all significantly correlations with community dissimilarity, with *R*^2^ values ranging from 0.46 to 0.83 (all *P* < 0.001) (Fig. 4b). These results indicate that taxonomic restructuring is tightly linked to concomitant shifts in microbial functional potential of the sediment microbiome.

**Fig 5:**
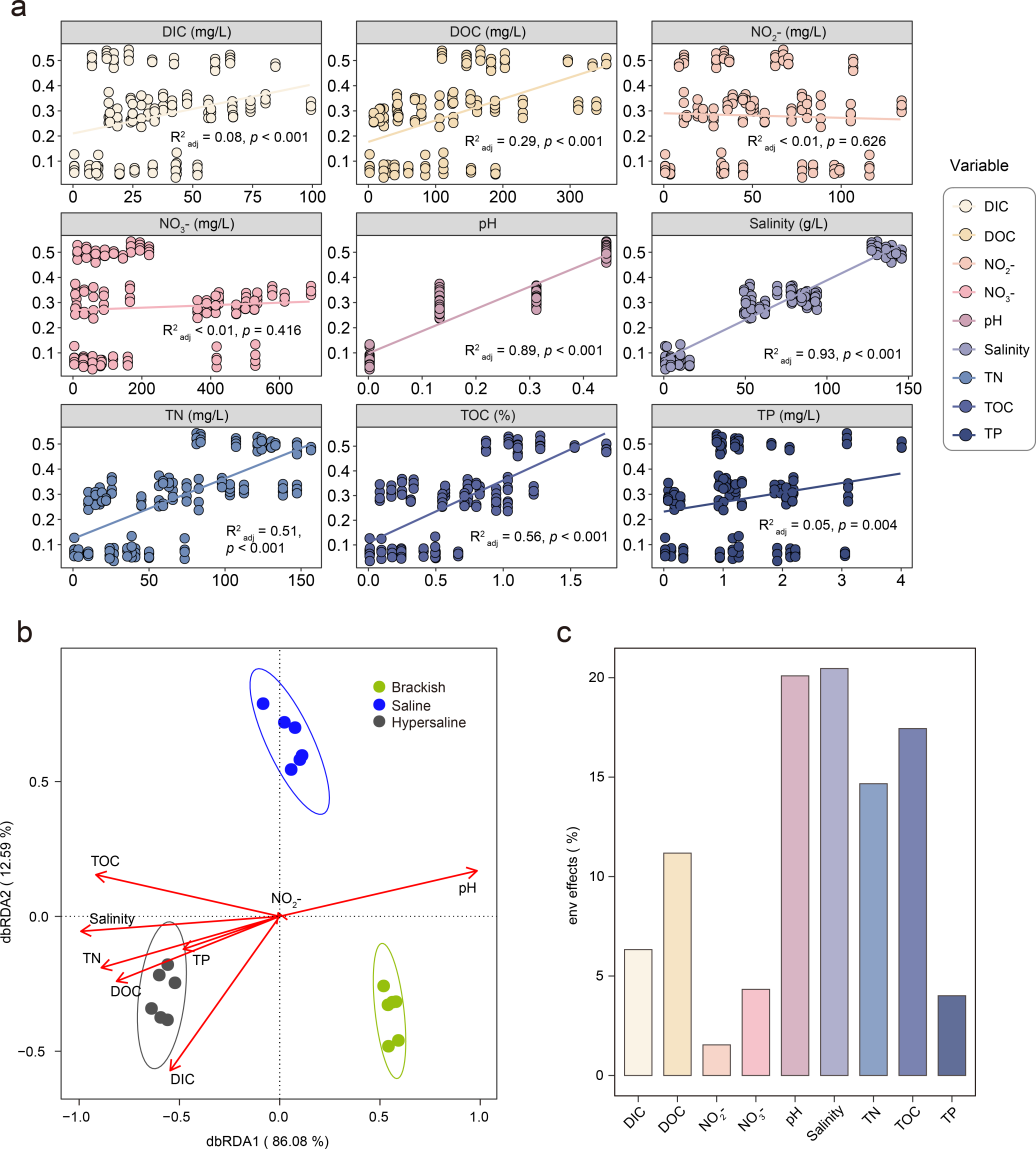
Influence of environmental factors on microbial functional gene structure. (**a**) Linear regression between CNPS functional gene distances and key physicochemical factors, identifying salinity as the dominant driver. (**b**) Distance-based redundancy analysis (dbRDA) ordination plot showing the separation of sediment samples along salinity and nutrient gradients, with vectors indicating influential environmental variables. (**c**) Variation partitioning analysis quantifying the unique and shared effects of salinity, nutrients, and other factors on functional gene composition.

The structuring of functional gene assemblages was also associated with major environmental variables, among which salinity was the strongest correlate. Linear regression indicated that DOC, TN, TOC, pH, and salinity were all correlated with overall functional gene profile distances (e.g., DOC: (*R*^2^_adj_ = 0.29, *P* < 0.001; salinity: (*R*^2^_adj_ = 0.93, *P* < 0.001)), while NO_2_^⁻^ showed no significant relationship (Fig. 5a). Distance-based redundancy analysis (dbRDA) corroborated this pattern, showing clear separation of brackish, saline, and hypersaline groups along axes primarily explained by salinity, TOC, and DIC (Fig. 5b). Variation partitioning analysis attributed the largest proportion of explainable variance in functional gene profiles to salinity, followed by pH, TN, and TOC (Fig. 5c), reinforcing its primary role among the measured variables.

The influence of both abiotic and biotic factors on functional potential was pathway-specific (Fig. S3). Salinity showed consistent correlations with gene abundances across most pathways (C degradation, C fixation, N cycling, P cycling, and S cycling). Other environmental variables, including TOC, DIC, DOC, and pH, showed specific associations with genes involved in carbon and nitrogen transformations. Furthermore, microbial diversity and community composition (PCoA axes) were positively correlated with the abundance of several functional genes, especially those involved in N and S cycling, highlighting the concomitant role of community structure. Together, these analyses demonstrate that functional gene profiles are jointly associated with salinity, local sediment geochemistry, and microbial community composition.

### Effects of salinity and microbial diversity on functional gene potential

**Fig 6:**
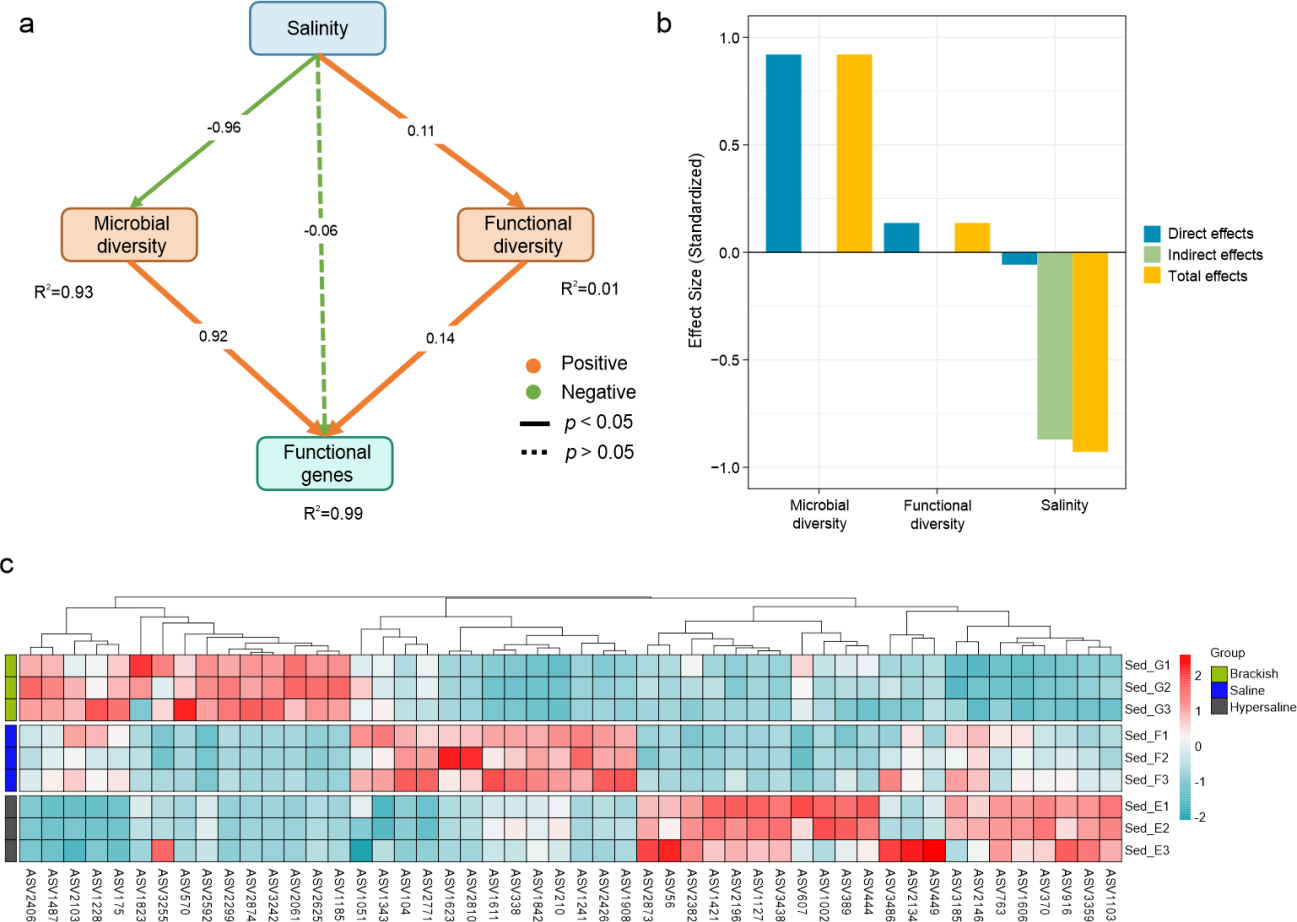
Effects of salinity and microbial diversity on functional gene potential. (**a**) Structural equation model (SEM) illustrating pathways through which salinity influences functional gene abundance, both directly and indirectly via microbial diversity. Microbial diversity exerted a strong positive effect on functional gene abundance. Arrow colors indicate positive (orange) or negative (green) effects, and line types represent significant (solid, *P* < 0.05) or non-significant (dashed) paths. (**b**) Standardized effect sizes of direct, indirect, and total effects of microbial diversity, functional diversity, and salinity on functional gene abundance. (**c**) Heatmap of ASV-level community composition across salinity groups, with clustering highlighting salinity-driven taxonomic shifts underlying functional changes.

Salinity was associated with changes in microbial functional gene potential through both direct and indirect pathways linked to microbial diversity and community composition. The structural equation model (Fig. 6a) showed a strong negative direct effect of salinity on microbial diversity (path coefficient = −0.96, *P* < 0.001) and a weaker positive direct effect on functional gene abundance (0.11). Microbial diversity, in turn, exhibited a strong positive direct effect on functional gene abundance (0.92). The model explained a high proportion of variance (93% for diversity, 99% for gene abundance), indicating a robust fit. Analysis of standardized effect sizes (Fig. 6b) quantified that the total effect of salinity on functional gene abundance was primarily mediated indirectly through its strong negative impact on microbial diversity, whereas its direct effect was comparatively minor. Functional gene diversity itself showed a negligible total effect.

Consistent with the model, an ASV-level heatmap revealed clear, salinity-dependent shifts in microbial community composition (Fig. 6c; detailed taxonomic assignments are provided in Table S3). Hierarchical clustering separated the samples into three distinct groups corresponding to brackish (Sed_G), saline (Sed_F), and hypersaline (Sed_E) sediments. Brackish samples were characterized by a set of ASVs that were highly abundant in this group but markedly declined at higher salinities. Saline sediments harbored a distinct set of ASVs that were preferentially enriched at intermediate salinity. In contrast, hypersaline samples were dominated by different ASVs that were prevalent under high-salinity conditions but rare or absent in the other groups.

## DISCUSSION

### Salinity-driven restructuring of microbial community and assembly mechanisms

Salinity-driven restructuring of microbial communities along the gradient highlights its role as a key environmental filter in sediment ecosystems (19, 56, 57). The successional shift in dominant lineages, from *Actinobacteriota*, *Gammaproteobacteria,* and *Alphaproteobacteria* dominance in low-salinity sediments toward *Gammaproteobacteria* and *Halobacteria* at higher salinities (Fig. 1a), indicates how increasing salinity strongly differentiates microbial community composition, consistent with patterns observed in other saline environments (12, 27). Such shifts are often attributed to osmotic stress, which imposes strong physiological constraints on microbial survival and niche partitioning (13, 58, 59).

Community assembly processes also varied with salinity, showing a nonlinear pattern in the relative influence of deterministic and stochastic forces (Fig. 1d). Stochastic processes, particularly ecological drift and dispersal limitation, were dominant at both salinity extremes. In contrast, the relative importance of deterministic selection (heterogeneous selection) was higher at moderate salinity although stochastic processes still prevailed overall. This pattern suggests that moderate salinity may intensify environmental filtering on taxa with divergent osmotic tolerances and metabolic strategies, whereas under both low and extremely high salinity conditions, historical contingencies (dispersal limitation) and demographic stochasticity (drift) become more influential (60, 61). These findings extend the current understanding by showing that salinity not only reshapes community composition but also modulates the balance between stochastic and deterministic assembly.

### Nonlinear functional responses and the link between taxonomic structure and biogeochemical potential

Microbial functional genes associated with biogeochemical cycling exhibited heterogeneous nonlinear responses to salinity (Fig. 2). The divergence in functional gene compositions among brackish, saline, and hypersaline sediments indicates that salinity was the key factor restructuring microbial metabolic potential (14, 62, 63), likely reflecting differences in the physiological tolerance and metabolic strategies required to maintain cellular homeostasis under varying osmotic conditions. The sensitivity to salinity, however, varied substantially among different metabolic pathways. Genes involved in carbon degradation, nitrogen cycling, and phosphorus cycling exhibited relatively weak but significant relationships with salinity, whereas those associated with carbon fixation, methane metabolism, and sulfur cycling showed nonlinear responses with salinity. This pathway-specific patterns suggest that key microbial metabolic functions differ in their sensitivity or adaptive response to salinity stress and the associated ionic environment. For example, processes like autotrophic carbon fixation pathways and methane metabolism are often catalyzed by specialized microbial guilds with relatively narrow ecological niches, which may make their genetic potential particularly sensitive to salinity variation (64, 65).

A notable pattern is the reduction in functional gene diversity observed in saline sediments, compared with brackish and hypersaline sediments (Fig. S2). One plausible interpretation for this pattern is the effect of intensified environmental filtering at intermediate salinity levels. Under elevated osmotic stress, microbial taxa may need to allocate a greater proportion of cellular resources to osmoregulation, potentially constraining the breadth of metabolic traits maintained within the community (66). Concurrently, the community may be in a transitional state where taxa adapted to lower salinity decline, while highly specialized halophiles have not yet become dominant, leading to a decrease in functional diversity (67). It is important to note that this study measured functional gene potential rather than expression or process rates; therefore, the ecological implications of this diversity pattern for biogeochemical functions remain to be tested.

The coupling between taxonomic structure and functional potential also varied among pathways. The weaker correlation observed for processes like carbon degradation suggests a degree of functional redundancy, where multiple taxa can perform similar metabolic roles, potentially buffering these functions against community turnover (12, 20, 58). In contrast, pathways including methane metabolism and nitrogen cycling appear to be more tightly linked to community composition, consistent with their frequent dependence on specialized microbial groups whose distributions are closely tied to specific ecological niches (64, 68). Consequently, salinity-driven changes in community structure may have a more direct and pronounced effect on the genetic potential of these specialized pathways.

### Salinity as the primary environmental driver and a conceptual framework

Among the measured environmental variables, salinity explained the largest proportion of variance in functional gene composition, surpassing other physicochemical factors such as TOC, DOC, DIC, and pH (Fig. 5). This is consistent with previous studies identifying ionic strength as a key constraint on microbial physiology and functional gene distribution in hypersaline systems (26, 58, 69). However, a significant portion of the variance was also explained by microbial community composition and diversity metrics, underscoring that functional gene assemblages are collectively associated with both abiotic conditions and biotic structure (20). The ASV-level heatmap (Fig. 6c) provides a taxonomic basis for this, illustrating that each salinity regime harbors a distinct microbial assemblage. The observed covariance between these compositional differences and the functional gene profiles is consistent with both properties being filtered by the salinity gradient, rather than evidencing direct causal interactions between specific taxa and functions.

**Fig 7:**
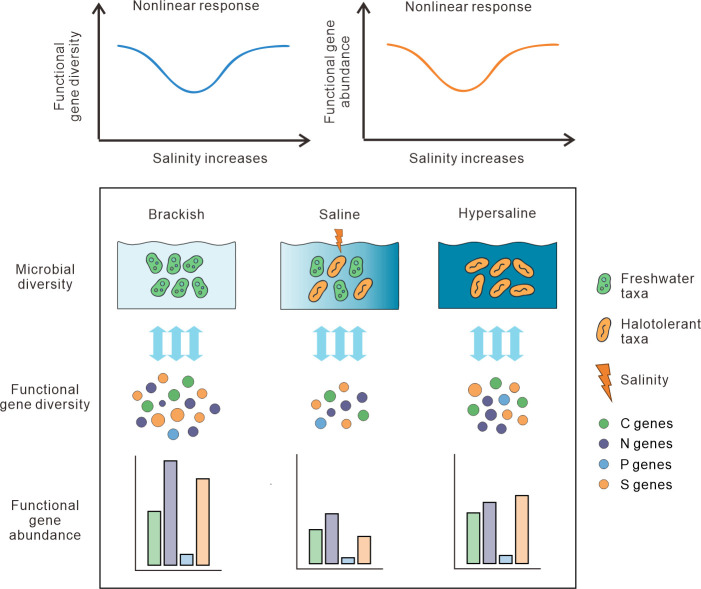
Aconceptual framework for salinity-driven nonlinear responses of microbial functional genes. The model illustrates how microbial composition, functional gene diversity, and functional gene abundance respond nonlinearly to salinity, with maximal suppression at moderate salinity due to combined osmotic stress and community transition.

The conceptual model (Fig. 7) developed in this study integrates these observations. In this framework, salinity acts as a primary environmental filter on microbial taxa, driving community turnover and influencing the balance of assembly processes. This taxonomic restructuring occurs in parallel with a reorganization of functional gene abundances, yielding the observed heterogeneous, pathway-specific responses in biogeochemical potential. Brackish sediments support taxonomically and functionally diverse communities. With increasing salinity, the community shifts toward more salt-tolerant lineages, accompanied by a measurable restructuring of functional traits and, as observed at intermediate salinity, a potential contraction in functional diversity. Under hypersaline conditions, specialized halophiles dominate, maintaining a distinct functional assemblage adapted to extreme osmotic stress. In summary, this study indicates that salinity regulates sediment biogeochemical potential through its coupled effects on environmental filtering, community composition, and functional gene inventory. Elucidating these interconnected relationships provides a framework for understanding how microbial communities mediate biogeochemical cycling across environmental gradients in saline lakes.

### Limitations of this study and recommendations for future studies

This study has several limitations that should be considered when interpreting the results. First, our sampling was restricted to a single season (early September 2024), providing only a temporal snapshot. Given the known intra-annual hydrological fluctuations of Yuncheng Salt Lake, characterized by evaporation-driven changes in water level and salinity, the microbial community and functional patterns we observed may not fully represent conditions across other seasons. Future seasonal or multi-year sampling is needed to evaluate the temporal stability of the patterns described here. Second, the quantification of microbial functional genes via the QMEC chip was limited to sediments with salinities up to ~158 g/L. Technical constraints, specifically challenges in DNA extraction and PCR amplification from near-saturation brines (>200 g/L), precluded the inclusion of the highest salinity samples. Therefore, our conclusions regarding salinity-driven patterns are constrained to the brackish-to-moderately hypersaline range and may not extend to the most extreme hypersaline conditions. Future methodological improvements are required to resolve functional gene profiles across the entire salinity spectrum. Finally, while we documented clear, salinity-associated shifts in functional gene *potential*, this study did not measure gene expression, process rates, or energy flows. Consequently, the direct ecological consequences of the observed genetic shifts for *in situ* biogeochemical cycling remain an open question. Integrating metatranscriptomic, rate measurement, and isotopic techniques in future work will be essential to link the genetic patterns identified here to actual ecosystem-scale processes.

### Conclusions

Salinity strongly restructures microbial communities in salt lake sediments and drives pronounced nonlinear responses in microbial functional genes involved in biogeochemical cycling. Shifts in dominant taxa and community assembly processes along the salinity gradient were closely associated with divergence in functional gene assemblages related to carbon, nitrogen, phosphorus, and sulfur transformations. Environmental analyses identified salinity as the principal driver of functional gene organization, while microbial diversity and community composition contributed important biotic controls. Together, these results demonstrate that salinity regulates sediment biogeochemical potential primarily through its coupled effects on microbial community structure and functional gene distributions.

## Data Availability

All 16S rRNA gene sequences generated in this study have been deposited in the NCBI Sequence Read Archive under project accession number PRJNA1346263 (SRA accession numbers: SAMN53475113–SAMN53475136).

## SUPPLEMENTAL MATERIAL

Supplemental material aem.00721-26-s0001.docx Fig. S1 to S3; Tables S1 to S3.

ASM does not own the copyrights to Supplemental Material that may be linked to, or accessed through, an article. The authors have granted ASM a non-exclusive, world-wide license to publish the Supplemental Material files. Please contact the corresponding author directly for reuse.
