# Supplementary material for: Salinity-driven nonlinear responses of microbial functional genes in sediment biogeochemical cycling across a salt lake gradient
Source: Appl Environ Microbiol. 2026 Jun 4;92(7):e00721-26. doi: 10.1128/aem.00721-26 (PMC13390440; doi:10.1128/aem.00721-26)
Supplement: Supplemental material — Fig. S1 to S3; Tables S1 to S3. [file aem.00721-26-s0001.docx]

**Supplementary material for**

**Salinity-driven nonlinear responses of microbial functional genes in sediment biogeochemical cycling across a salt lake gradient**

Mingxian Han^1^, Jianrong Huang^1^*, Jian Yang^1^, Qing Liu^1^, Chuanxu Wang^2,3^, Xin Li^4^, Hongchen Jiang^1^*

^1^School of Life Sciences, Henan University, Kaifeng 475004, China.

^2^College of Life Sciences, Yuncheng University, Yuncheng 044000, China.

^3^Shanxi Key Laboratory of Yuncheng Salt Lake Ecological Protection and Resource Utilization, Yuncheng University, Yuncheng 044000, China.

^4^Department of Biology, Xinzhou Normal University, Xinzhou 034000, China.

*Correspondence:

Jianrong Huang, huangjianrong@henu.edu.cn

Hongchen Jiang, Jiangh@henu.edu.cn

**Fig.S1** Sampling locations of sediment samples from Yuncheng Salt Lake, Shanxi Province, China. Note that samples sed-C-yellow and sed-C-purple were collected from the same pond but exhibited distinct coloration (yellow and deep purple, respectively).

**
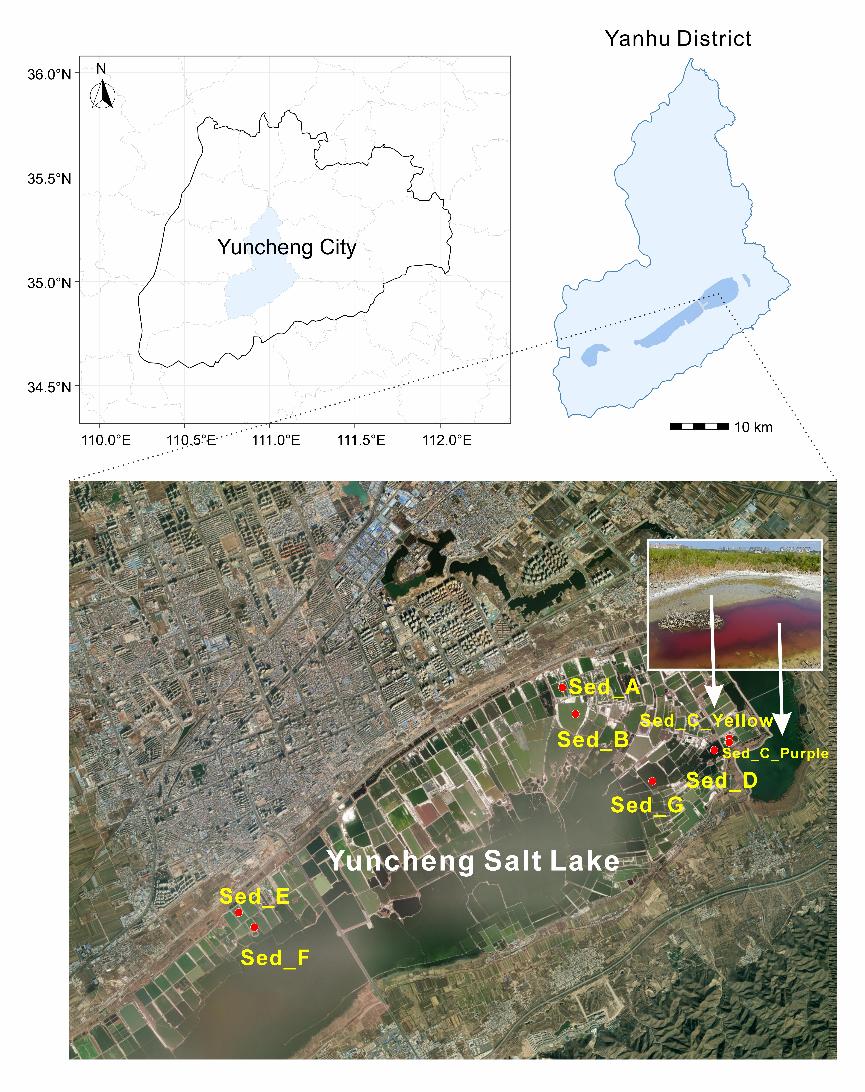
**

**Fig. S2** Salinity-driven variations in Shannon diversity (a) and observed richness (b) of functional gene (involved in C, N, P, and S cycling) across brackish, saline, and hypersaline sediment groups.


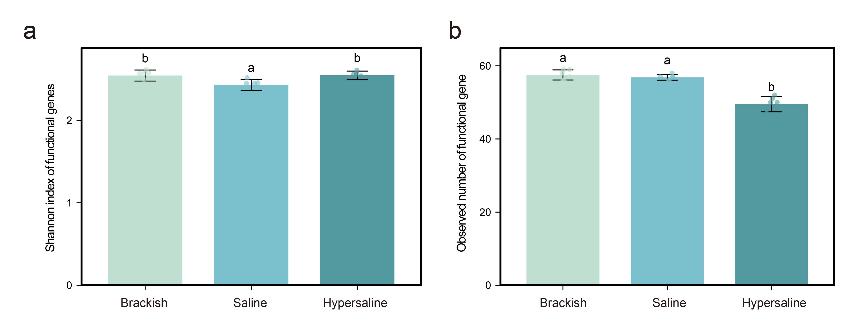


**Fig. S3** Abiotic and biotic determinants shaping functional gene profiles across major biogeochemical pathways. Spearman correlations between biological factors and environmental variables with gene abundances associated with carbon degradation, carbon fixation, methane metabolism, nitrogen cycling, phosphorus cycling and sulfur cycling. Circle size denotes the absolute correlation coefficient (|r|), and circle color indicates the direction and magnitude of the correlation. Dark blue represents stronger positive correlations, and dark red represents stronger negative correlations. Statistical significance is denoted by asterisks: ****p* < 0.001, ***p* < 0.01, **p* < 0.05.


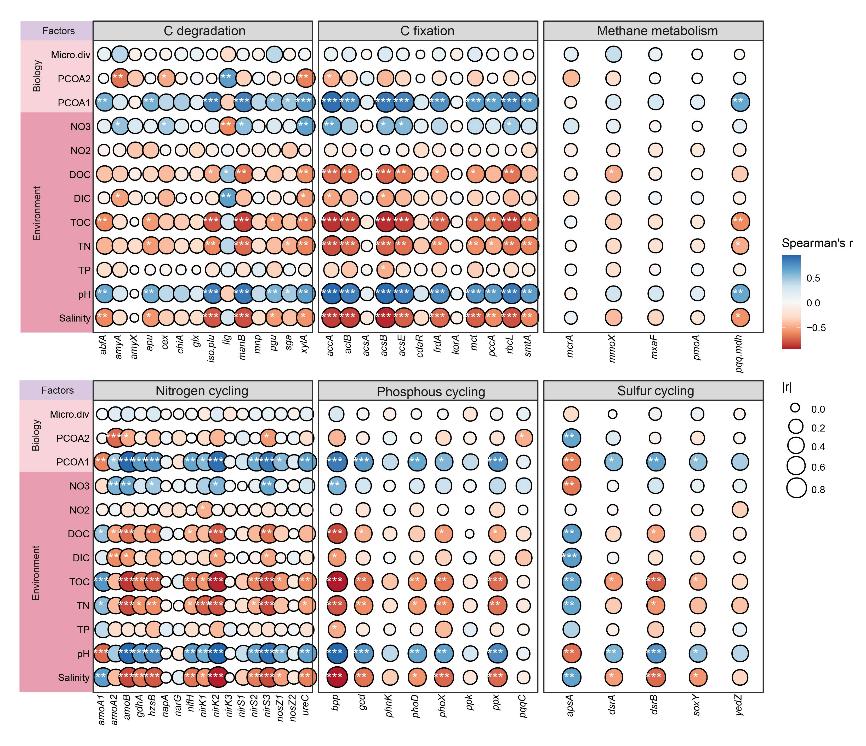


**Table S1** Physicochemical parameters of sediments across all sampling sites.

| Samples | Salinity (g/L) | pH | TP (mg/L) | TN (mg/L) | TOC (%) | DIC  (mg/L) | DOC (mg/L) | NO_2_^-^(mg/L) | NO_3_^-^(mg/L) |
| --- | --- | --- | --- | --- | --- | --- | --- | --- | --- |
| Sed_A1 | 163.54 | 8.10 | 2.02 | 116.35 | 2.07 | 253.90 | 467.32 | 300.00 | 607.68 |
| Sed_A2 | 176.26 | 8.10 | 3.69 | 112.06 | 2.14 | 241.85 | 407.67 | 204.24 | 912.54 |
| Sed_A3 | 176.32 | 8.10 | 2.92 | 71.79 | 1.91 | 174.71 | 218.23 | 77.64 | 704.22 |
| Sed_B1 | 133.83 | 7.72 | 2.07 | 119.40 | 1.38 | 238.84 | 363.58 | 235.86 | 823.38 |
| Sed_B2 | 129.73 | 7.72 | 3.07 | 132.39 | 1.78 | 244.00 | 334.71 | 118.98 | 778.56 |
| Sed_B3 | 141.58 | 7.72 | 4.15 | 176.35 | 2.79 | 289.27 | 537.84 | 96.24 | 713.22 |
| Sed_C_Pink1 | 259.60 | 8.18 | 2.87 | 110.12 | 1.08 | 235.01 | 281.46 | 214.32 | 825.48 |
| Sed_C_Pink2 | 255.53 | 8.18 | 4.25 | 97.71 | 1.35 | 225.18 | 236.27 | 118.80 | 902.70 |
| Sed_C_Pink3 | 253.66 | 8.18 | 4.81 | 99.56 | 1.12 | 230.31 | 255.88 | 96.48 | 808.92 |
| Sed_C_Yellow1 | 202.98 | 8.21 | 2.23 | 83.80 | 0.79 | 211.35 | 173.22 | 195.66 | 831.06 |
| Sed_C_Yellow2 | 200.52 | 8.21 | 2.87 | 66.26 | 0.79 | 162.86 | 113.56 | 92.70 | 810.12 |
| Sed_C_Yellow3 | 199.64 | 8.21 | 3.33 | 62.39 | 0.83 | 169.82 | 142.67 | 64.38 | 820.22 |
| Sed_D1 | 258.96 | 8.55 | 1.27 | 101.90 | 1.04 | 224.30 | 256.27 | 166.38 | 826.08 |
| Sed_D2 | 250.94 | 8.55 | 5.07 | 130.51 | 2.36 | 300.97 | 400.97 | 130.86 | 683.82 |
| Sed_D3 | 243.37 | 8.55 | 5.27 | 162.33 | 2.50 | 300.08 | 485.70 | 108.12 | 726.00 |
| Sed_E1 | 158.95 | 8.18 | 1.12 | 188.83 | 1.45 | 222.46 | 248.88 | 182.46 | 827.40 |
| Sed_E2 | 150.08 | 8.18 | 3.22 | 148.76 | 1.61 | 228.72 | 212.34 | 87.12 | 671.94 |
| Sed_E3 | 165.12 | 8.18 | 4.15 | 197.98 | 2.10 | 247.65 | 399.51 | 84.24 | 748.08 |
| Sed_F1 | 72.00 | 8.49 | 1.06 | 124.12 | 1.37 | 191.63 | 171.97 | 164.22 | 833.64 |
| Sed_F2 | 73.05 | 8.49 | 2.20 | 51.60 | 1.29 | 181.62 | 89.24 | 49.08 | 1246.98 |
| Sed_F3 | 81.37 | 8.49 | 2.30 | 86.47 | 0.89 | 148.84 | 62.41 | 132.48 | 1359.24 |
| Sed_G1 | 20.08 | 8.62 | 0.16 | 68.36 | 0.35 | 205.97 | 47.65 | 153.72 | 836.46 |
| Sed_G2 | 20.19 | 8.62 | 2.05 | 42.37 | 0.35 | 163.65 | 68.19 | 76.44 | 889.14 |
| Sed_G3 | 23.56 | 8.62 | 2.35 | 65.65 | 0.58 | 215.14 | 104.96 | 120.36 | 862.38 |

**Table S2** AIC comparison between linear and loess models for the relationship between salinity and functional gene abundance in six biogeochemical pathways.

| Pathway | Linear model AIC | Loess model AIC | ΔAIC |
| --- | --- | --- | --- |
| Carbon fixation | 7590.45 | 6976.05 | 614.40 |
| Methane metabolism | 2476.87 | 2221.31 | 255.56 |
| Carbon degradation | 9458.80 | 8535.54 | 923.26 |
| Nitrogen cycling | 13923.55 | 12796.26 | 1127.29 |
| Phosphorus cycling | 5133.01 | 4570.13 | 462.88 |
| Sulfur cycling | 3280.40 | 3105.52 | 264.88 |

**Table S3** Taxonomic classification information of differential ASVs across brackish, saline, and hypersaline sediment groups (corresponding to Fig. 6c).

| ASV | Phylum | Class | Order | Family |
| --- | --- | --- | --- | --- |
| ASV2046 | *Bacteroidota* | *Bacteroidia* | *Bacteroidales* | *Prevotellaceae* |
| ASV1487 | *Actinobacteriota* | *Actinobacteria* | *Micrococcales* | *Microbacteriaceae* |
| ASV2103 | *Actinobacteriota* | *Actinobacteria* | Unclassified | Unclassified |
| ASV1228 | *Actinobacteriota* | *Actinobacteria* | *Nitriliruptorales* | *Nitriliruptoraceae* |
| ASV175 | *Proteobacteria* | *Gammaproteobacteria* | Unclassified | Unclassified |
| ASV1823 | *Bacteroidota* | *Bacteroidia* | *Flavobacteriales* | *Flavobacteriaceae* |
| ASV3255 | *Proteobacteria* | *Gammaproteobacteria* | *Nitrococcales* | *Nitrococcaceae* |
| ASV570 | *Proteobacteria* | *Gammaproteobacteria* | *Coxiellales* | *Coxiellaceae* |
| ASV2592 | *Bacteroidota* | *Rhodothermia* | *Balneolales* | *Balneolaceae* |
| ASV2299 | *Actinobacteriota* | *Actinobacteria* | *Micrococcales* | *Microbacteriaceae* |
| ASV2874 | *Proteobacteria* | *Gammaproteobacteria* | Unclassified | Unclassified |
| ASV3242 | *Actinobacteriota* | *Actinobacteria* | *Micrococcales* | *Microbacteriaceae* |
| ASV2061 | *Proteobacteria* | *Gammaproteobacteria* | *Burkholderiales* | MWH-UniP1_aquatic_group |
| ASV2625 | *Actinobacteriota* | *Actinobacteria* | *PeM15* | *Unclassified* |
| ASV1185 | *Bacteroidota* | *Rhodothermia* | *Balneolales* | *Balneolaceae* |
| ASV1051 | *Actinobacteriota* | *Actinobacteria* | *PeM15* | *Unclassified* |
| ASV1343 | *Proteobacteria* | *Alphaproteobacteria* | *Rhodobacterales* | *Rhodobacteraceae* |
| ASV104 | *Actinobacteriota* | *Acidimicrobiia* | *Microtrichales* | *Ilumatobacteraceae* |
| ASV2771 | *Bacteroidota* | *Rhodothermia* | *Balneolales* | *Balneolaceae* |
| ASV1623 | *Proteobacteria* | *Alphaproteobacteria* | *SAR11_clade* | *Clade_I* |
| ASV2810 | *Proteobacteria* | *Alphaproteobacteria* | *Rhodospirillales* | AEGEAN-169_marine_group |
| ASV1611 | *Deinococcota* | *Deinococci* | *Deinococcales* | *Trueperaceae* |
| ASV338 | *Deinococcota* | *Deinococci* | *Deinococcales* | *Trueperaceae* |
| ASV1842 | *Bacteroidota* | *Rhodothermia* | *Balneolales* | *Balneolaceae* |
| ASV210 | *Actinobacteriota* | *Actinobacteria* | PeM15 | Unclassified |
| ASV1241 | *Proteobacteria* | *Alphaproteobacteria* | SAR11_clade | Clade_III |
| ASV2426 | *Proteobacteria* | *Gammaproteobacteria* | Unclassified | Unclassified |
| ASV1908 | *Actinobacteriota* | *Actinobacteria* | *Micrococcales* | *Microbacteriaceae* |
| ASV2873 | *Proteobacteria* | *Gammaproteobacteria* | *Nitrococcales* | *Nitrococcaceae* |
| ASV56 | *Bacteroidota* | *Bacteroidia* | *Flavobacteriales* | *Crocinitomicaceae* |
| ASV2382 | *Bacteroidota* | *Bacteroidia* | *Flavobacteriales* | *Cryomorphaceae* |
| ASV1421 | *Actinobacteriota* | *Actinobacteria* | *Micrococcales* | *Microbacteriaceae* |
| ASV2196 | *Proteobacteria* | *Gammaproteobacteria* | Unclassified | Unclassified |
| ASV1127 | *Firmicutes* | *Bacilli* | *Izemoplasmatales* | *Izemoplasmataceae* |
| ASV3438 | *Deinococcota* | *Deinococci* | *Deinococcales* | *Trueperaceae* |
| ASV607 | *Proteobacteria* | *Gammaproteobacteria* | *Nitrococcales* | *Nitrococcaceae* |
| ASV1002 | *Halobacterota* | *Halobacteria* | *Halobacterales* | *Haloferacaceae* |
| ASV389 | *Firmicutes* | *Bacilli* | *Izemoplasmatales* | uncultured_bacterium |
| ASV444 | *Bacteroidota* | *Bacteroidia* | *Flavobacteriales* | *Flavobacteriaceae* |
| ASV3486 | *Proteobacteria* | *Gammaproteobacteria* | *Pseudomonadales* | *Litoricolaceae* |
| ASV2134 | *Proteobacteria* | *Gammaproteobacteria* | *Burkholderiales* | *Alcaligenaceae* |
| ASV449 | *Proteobacteria* | *Gammaproteobacteria* | *Nitrococcales* | *Nitrococcaceae* |
| ASV3185 | *Proteobacteria* | *Gammaproteobacteria* | Unclassified | Unclassified |
| ASV2146 | *Actinobacteriota* | *Actinobacteria* | PeM15 | Unclassified |
| ASV763 | *Actinobacteriota* | *Actinobacteria* | *Nitriliruptorales* | *Nitriliruptoraceae* |
| ASV1606 | *Actinobacteriota* | *Actinobacteria* | PeM15 | Unclassified |
| ASV370 | *Actinobacteriota* | *Actinobacteria* | *Micrococcales* | *Microbacteriaceae* |
| ASV916 | *Bacteroidota* | *Bacteroidia* | *Flavobacteriales* | *Cryomorphaceae* |
| ASV3359 | *Bacteroidota* | *Rhodothermia* | *Balneolales* | *Balneolaceae* |
| ASV1103 | *Actinobacteriota* | *Actinobacteria* | *Micrococcales* | *Microbacteriaceae* |
